# “Can you hear me now?”: a qualitative exploration of communication quality in virtual primary care encounters for patients with intellectual and developmental disabilities

**DOI:** 10.1186/s12875-023-02055-z

**Published:** 2023-04-20

**Authors:** Avra Selick, Janet Durbin, Yani Hamdani, Jennifer Rayner, Yona Lunsky

**Affiliations:** 1 Institute for Health Policy, Management and Evaluation, University of Toronto, Toronto, ON Canada; 2 Azrieli Adult Neurodevelopmental Centre, Centre for Addiction and Mental Health, Toronto, ON Canada; 3 Provincial System Support Program, Centre for Addiction and Mental Health, Toronto, ON Canada; 4 Department of Psychiatry, University of Toronto, Toronto, ON Canada; 5 Department of Occupational Science & Occupational Therapy, University of Toronto, Toronto, ON Canada; 6 Centre for Studies in Family Medicine, Western University, London, ON Canada; 7 Department of Research and Evaluation, Alliance for Healthier Communities, Toronto, ON Canada

**Keywords:** Communication, Virtual health care, Qualitative, Intellectual and developmental disabilities, Primary care

## Abstract

**Background:**

High quality communication is central to effective primary care. The COVID-19 pandemic led to a dramatic increase in virtual care but little is known about how this may affect communication quality. Adults with intellectual and developmental disabilities (IDD) can experience challenges communicating or communicate in non-traditional ways. This study explored how the use of virtual modalities, including telephone and video, affects communication in primary care interactions for patients with IDD.

**Methods:**

This qualitative descriptive study included semi-structured interviews with a multi-stakeholder sample of 38 participants, including 11 adults with IDD, 13 family caregivers, 5 IDD support staff and 9 primary care physicians. Interviews were conducted in Ontario, Canada between March and November 2021 by video-conference or telephone. A mixed inductive and deductive thematic analysis approach was used to code the data and identify themes. Themes were reviewed and refined with members of each stakeholder group.

**Results:**

Four elements of communication were identified that were affected by virtual care: (1) patient engagement in the virtual appointment; (2) the ability to hear other participants and have the time and space to be heard; (3) the ability to use nonverbal communication strategies; and (4) the ability to form trusting relationships. In some cases, the virtual platform hindered these elements of communication. Video offered some advantages over telephone to support nonverbal communication, and stimulate engagement; though this could be limited by technical challenges. For adults with IDD who find it difficult to attend in-person appointments, virtual care improved communication quality by allowing them to participate from a space where they were comfortable.

**Conclusion:**

Though there are circumstances in which virtual delivery can improve communication for patients with IDD, there are also challenges to achieving high quality patient-provider communication over telephone and video. Improved infrastructure and training for providers, patients and caregivers can help improve communication quality, though in some cases it may never be appropriate. A flexible patient-centred approach is needed that includes in-person, telephone and video options for care.

## Background

Virtual health care, including care delivered by telephone and video, has been available for decades but until recently accounted for only a very small proportion of health care use. This changed during the COVID-19 pandemic when much of health care, including primary care, abruptly shifted to virtual delivery around the world [1–3]. This significant shift in care has provided a unique opportunity to learn about the value and appropriateness of virtual care for different patient populations.

One concern related to virtual care is the implications for quality of communication between patients and health care providers [4–6]. Communication is the foundation of the clinical interaction. High quality communication is necessary for providers to obtain accurate histories and symptom descriptions from patients and caregivers, and for patients and caregivers to understand treatment instructions [7, 8]. Positive communication can also build the confidence, comfort and trust necessary for patients to disclose health information [9, 10]. Poor communication can lead to misdiagnosis, inappropriate treatment, poor treatment adherence, low patient satisfaction and poor health outcomes [7–10]. Traditional strategies to facilitate high quality communication include making eye contact, observing and utilizing body language, using visual aids or demonstrations, and using physical touch to connect with patients [8, 10]. The increased prevalence of virtual care raises the question of whether the quality of communication achieved in-person can be replicated virtually when many of these strategies are not possible. The answer to this question may differ for different patient groups.

People with intellectual and developmental disabilities (IDD) are a group that requires additional consideration to ensure health care quality is not compromised. For the purpose of this study, IDD was broadly defined to include any conditions of childhood-onset that impact cognitive and adaptive functioning across the lifespan [11]. This includes, for example, intellectual disabilities, autism, Down syndrome, and fetal alcohol spectrum disorders. People with IDD have a long history of being mistreated, neglected and underserved by health care systems [12, 13]. Compared with people without IDD, they continue to be less likely to receive quality care and more likely to experience poor health outcomes including lengthy and repeated hospitalization and premature mortality [12, 14, 15].

Poor communication in health care settings has been identified as one of the most significant barriers to quality care for patients with IDD [16]. Communication is a dynamic interaction between two or more participants that always includes a message, a sender of the message, and a receiver of the message. Adaptations and accommodations may be necessary in any part of this interaction to achieve effective communication. Patients with IDD may communicate and process information differently than patients without IDD [16–18]. Some patients with IDD may be nonspeaking or less able to speak in certain situation and they may use other strategies to communicate. Effectively communicating with these patients may require the use of augmentative and assistive communication devices, sign language, easy read information, written communication or communicating with the support of a caregiver [18, 19]. In numerous studies, health care providers across jurisdictions and disciplines have reported that they lacked skills and training on how to adjust their approach to support effective communication with this patient group [16, 19–21]. Indeed, studies in the US, Canada and Australia found that IDD receives little attention in undergraduate and residency medical education, and most of what is taught focuses on children as opposed to adults [22–24]. With the rise of virtual care, it is important that the needs of this population are considered so that existing challenges are not exacerbated.

Prior research focused on the general population has suggested that communication in video-based health care visits can be just as effective or even have advantages over in-person visits [25–29]. It may simply require that providers adjust their communication style, such as through greater verbalization of empathy and understanding, using exaggerated gestures and expressions, and asking more clarifying questions [25]. In these studies, some patients found video interactions less intimidating; they felt a greater sense of control and were more comfortable discussing difficult topics [25, 30]. Video can also replicate the benefits of home visits in which providers see patients in their home environments, thus supporting a greater understanding of the patient and a stronger relationship [30].

Much of this research, however, was conducted prior to the pandemic in a very different context. Providers in these studies often had experience delivering virtual care and patients typically chose to opt into virtual care. Patients were also often required to meet certain prerequisites (e.g., access to technology) that would promote successful encounters. Prior research was largely focused on video-based care, while the majority of virtual care during the pandemic has been delivered by telephone [31, 32]. Some of the early pandemic research on virtual care, when neither patients nor providers were prepared for this new mode of delivery, reported that the quality of the patient-provider interaction was sometimes compromised due to lack of body language, facial cues and physical touch and given the distractions that can be present for patients and providers participating from their homes [33–36].

We identified few previous studies on communication with adults with IDD during virtual health care interactions and they reported mixed findings. Harris and colleagues [37] interviewed autistic adults and their family members during the pandemic and found that video-based primary care visits generally supported similar or improved communication as it removed patients from overstimulating office environments. However, some patients were more distracted when in their home environment and less engaged in the visit. Adams and colleagues [38] surveyed clinicians delivering telemental health care (including by telephone and video) to autistic people during the pandemic and found that communication was one of the most commonly identified challenges. Sehlin and colleagues [39] evaluated an internet-based support and coaching model for autistic youth prior to the pandemic and found that asynchronous written communication was perceived by some youth to be more accurate, thereby reducing misunderstandings and memory issues. Though other youth disliked text-based communication and felt the interaction quality was negatively impacted by the lack of nonverbal communication. These were all small studies focused on autistic individuals who may have different experiences than other individuals with IDD.

This aim of this paper was to explore how the use of virtual modalities affects communication between patients with IDD and primary care providers. Study findings can inform clinical practice, including identification of when virtual care is appropriate for patients with IDD. These findings may also be relevant across different health care settings and for other populations with communication or cognitive challenges. This paper is part of a larger qualitative study on the experiences of virtual primary care for adults with IDD [40].

## Methods

### Study design

The study design was guided by qualitative description methodology which focuses on describing and understanding participant experiences with the goal of achieving descriptive and interpretive validity [41–43]. Qualitative description is a pragmatic, participant-centred approach recommended for applied health services research aimed at informing policy and practice such as the present study [44]. This study was conducted as part of the doctoral thesis of the first author and was supported by a team that included researchers working in both the health care and IDD sectors, some of whom are also family members of people with IDD.

### Data collection

We conducted semi-structured interviews with adults with IDD, caregivers (including family members and paid staff), and primary care physicians. The study was restricted to participants living in Ontario, Canada who had experience participating in at least one virtual health care visit for a patient with IDD over age 18. Virtual visits were defined as any health care visit conducted remotely, including by video and telephone. Given the limited prior research on this topic, we used a maximum variation sampling strategy [45] to achieve a diverse study sample considering age, gender, living situation (i.e., with family, in a supported setting or independently), and geographic location. To achieve these aims, we used broad recruitment strategies utilizing existing health care provider and self-advocate networks, social media, existing research databases and snowball strategies. Additional targeted recruitment was conducted as needed.

All interviews were conducted by the first author who had 10 years of qualitative research experience, including prior experience conducting interviews with adults with IDDs. The interview guide was informed by a previous scoping review of the literature [46] and the Levesque Access to Care Framework [47]. The interview guide focused on the experience of receiving, supporting or delivering virtual care, with a subset of questions specific to communication. These questions were adapted to be appropriate for different participants but focused on participation (e.g., To what extent were your patients with IDD included in virtual appointments?), communication quality (e.g., Were you able to understand the doctor when you talked on the phone?), comfort interacting virtually (e.g., Did you like talking with the doctor on the phone?), and relevance of context (e.g., Under which circumstances was communication more or less successful?). Demographic information on age, gender, disability, and geographic location was also collected.

The interviews were conducted between March and November 2021. Due to restrictions related to the COVID-19 pandemic, the interviews were conducted by telephone or through video conferencing according to participate preference. All interviews were conducted in English. Interviews lasted approximately 20–60 min and were audio-recorded and transcribed. Field notes were taken during and immediately following each interview to document interviewer impressions [48]. Adults with IDD had the option of being interviewed independently or with a support person. An honorarium was provided to all participants. The study was approved by the research team’s Institutional Research Ethics Board, and all participants provided informed consent prior to participating in the study.

### Analysis

A mixed inductive and deductive thematic analysis approach was used to guide the analysis [49, 50]. Coding was informed by previous literature on virtual care [46] but remained relatively open and data driven. The first author (AS) developed an initial codebook based on a review of all transcripts and field notes. A subset of transcripts from each stakeholder group were reviewed and discussed with two additional authors (JD and YL) to identify key ideas and patterns of ideas and refine the initial codebook. Using Nvivo 12 software, the first author (AS) then coded all transcripts, iteratively updating and refining the codebook throughout the process. A concept mapping exercise was conducted to visually explore relationships and patterns across all codes. Multiple maps were created in which codes were grouped in different ways. These initial maps were reviewed and discussed by all the study authors and key themes were identified. These preliminary themes were then reviewed, discussed and refined with members of each stakeholder group (i.e., self-advocates, caregivers and family physicians) as part of a peer debriefing process [51]. This paper reports on the subset of themes related to communication. Quotations are included to illustrate findings.

## Results

### Participants

This study included 38 participants: 11 adults with IDD, 13 family members (8 parents and 5 siblings), 5 IDD support staff and 9 primary care physicians. Participants included 25 women and 13 men, between 23 and 69 years old, living across Ontario. There were an additional 21 individuals who inquired about the study but either were ineligible or did not follow up to schedule an interview. See Table 1 for participant characteristics. In two cases, individuals with IDD were interviewed together with a family member and in one case two family members were interviewed together. Seven of the nine participating primary care physicians reported having practices with a particular focus on patients with IDD; the remaining two physicians had experience providing virtual care to patients with IDD but no particular focus on this patient group.

**Table 1:** Participant characteristics

Stakeholder group	Adults with IDD	Family members	Paid staff	Health care providers
**Total**	11	13	5	9
**Age range**				
18–34	6	0	2	3
35–50	4	2	2	5
51–65	0	7	1	1
66+	1	4	0	0
**Gender**				
Men	4	3	3	3
Women	7	10	2	6
**Location**				
Greater Toronto Area	5	8	1	5
Eastern Ontario	2	2	2	3
Western Ontario	4	3	0	1
Northern Ontario	0	0	2	0
**Living situation of the participant with IDD or the adult/s with IDD supported by the participant**				
Family	6	11	1	n/a
Supported	1	2	3	n/a
Independent	4	0	1	n/a
Disability of the participant with IDD or the adult/s with IDD supported by the participant*				
Autism (with or without an intellectual disability)	6	7	1	n/a
Intellectual disability	5	2	0	n/a
Down syndrome	0	4	0	n/a
Multiple	n/a	n/a	4	9

*Note that disabilities were recorded based on self-report and were not directly assessed

### Themes

We identified four main themes related to communication: (1) Participation and engagement; (2) Hearing and being heard; (3) Seeing and being seen; and (4) Connection and trust.

#### Participation and engagement

The first theme was related to how virtual care affected the level and quality of patient participation in the health encounter, a necessary prerequisite for communication. Patients with IDD were sometimes less engaged or did not participate at all in health care visits conducted remotely. One challenge, as will be discussed further under theme 4 (connection and trust), was that some people with IDD do not engage in the same way when they are not the same space with the other person. The father of a 25 year old autistic man noted that his son would have no interest in participating in a telephone appointment. He explained: “*Cognitively, he… he’s more interested in stuff that he can touch and feel. […] I mean, it’s not like he doesn’t know how to use the phone, but it just wouldn’t have any value to him*.”

A second challenge was it can be easier for patients to become distracted or wander off when participating in virtual visits. Patients might become distracted by objects or other activities in their homes. For example, the sister of a 55 year old woman with an intellectual disability shared: “*I think because there’s too many distractions at home. […] I remember another time she was washing the dishes. She was making all this noise. ‘I’m busy. I got things to do.’ So if she’s there in person, she doesn’t have to do those things*.” Some people with IDD also found it very distracting if there were any challenges with the technology, for example a flickering video or out-of-sync audio.

Participants noted that video was often a better option than telephone for keeping people engaged. The brother of a 55 year old man with Down syndrome explained that on the telephone his brother “*might not register very much what’s going on, he might not even realize who you are, so he depends on the visual to identify you.* […] *We can engage him with visuals on a video call much more easily.*” Phone calls were much more likely to take place between the caregiver and the health care provider and not include the patient, leaving the patient with no opportunity to communicate at all.

There were, however, some participants who found it easier to participate in remote visits. Participants who become very anxious or agitated when travelling to a health care visit or waiting in the waiting room found it easier to be fully engaged in health care visits that occurred in the comfort of their own homes. The mother of a 34 year old woman with Down syndrome explained: “*Because of her social anxiety and the vibes that she gets, she’s very sensitive to everybody’s vibes around her. I think that she would have an easier time communicating [virtually] because she’s just going to…she’s in a safe, known space and she’s dealing just one on one without all the background stuff that happens in a physical setting.”* Additionally, when the patient was less agitated, it was also easier for the caregiver to fully engage in the health care visit. An IDD support staff shared, “*I feel the appointment is better because the individual is relaxed, mom and I are more relaxed. So mom and I can focus on giving the information to the doctor.”* Effective communication was contingent on selecting the modality that best supported participation and engagement in the health care interaction, which varied for different patients and caregivers.

#### Hearing and being heard

The second theme was related to participants’ ability to clearly hear other individuals in the health encounter and to be heard in return. Hearing other participants was sometimes challenging in phone encounters, especially for people with hearing impairments or if other individuals had an unfamiliar accent. The mother of a 28 year old man with Down syndrome shared: “*He has some hearing problems so…that’s definitely an issue with the phone calls […] His psychiatrist has a very strong accent so it’s challenging enough when we’re in person with her but there’s a lot of, ‘What did you say? What did you say?’ when we’re on the phone.*” A 35 year old woman with an intellectual disability and a hearing impairment, shared, “*Because of my hearing, I would prefer video so I could see the person but some offices don’t allow that*.” Hearing could also be challenging in video encounters due to the patient, caregiver or provider’s lack of technical proficiency (e.g., inappropriate placement of the microphone or inability to adjust the volume). A staff member at an IDD agency explained: “*I think sometimes [staff] miss pieces because […] either they’re not hearing properly or they’re overwhelmed with the call itself, the technology piece, or they don’t know how to turn their speakers up, [or they] will have notes right in front of a speaker and you can’t hear what they’re saying because it’s that chchch sound.”*

Another related challenge was ensuring everyone was heard and had the opportunity to speak when there were more than two people participating in the virtual visit. Participants shared that particularly on telephone calls but even on video calls it could be challenging to navigate conversations given the lack of visual cues or technology lags that can cause multiple people to speak at once or find it difficult to interject. A staff member at an IDD agency explained: “*I think the big issue is sort of the dead air. When you have a lot of people on there, one person goes to talk, and another person goes to talk and then nobody talks, and then like, ‘oh I’m just not going to ask’. It happens at every appointment.*” Additionally, some people with IDD produce vocalizations or other sounds that can make it challenging to hear other participants. As one physician shared: “*Some of my patients have a lot of vocalizations and the microphone would just pick those up in a way that was really, really loud. […] It would almost be impossible to hear.”* During in-person visits it was possible to talk more loudly to compensate or step away if needed, but this was not possible virtually.

Despite these challenges, participants also identified the value of virtual care for supporting alternative ways of being heard. Virtual appointments could potentially make it easier to include interpreters if interpretation services are not be available locally. Some video platforms allow participants to type their responses which was an easier and more comfortable way to communicate than verbalizing for some individuals.

#### Seeing and being seen

The third theme was related to the importance of having a visual to support effective communication. Participants suggested that nonverbal communication, including body language and facial expressions, is particularly important for people with IDD to support comprehension. Video was generally seen as a better option than telephone, as it allowed for at least some nonverbal communication. A 43 year old autistic woman explained: “*Video actually would be good because then I can gauge some facial reactions. I may be bad at it, but I can still gauge some. It gives me a sense of how someone is reacting to what I’m saying*.” However, some participants felt that even video was not an adequate substitute for in-person communication. A 48 year old autistic man explained: *“I think when we miss half the body language to begin with, not having the in-person is too much of a disadvantage. Because even on video, you miss some body language.”*

Participants noted that nonverbal communication was also important for health care providers to understand their patients with IDD, especially patients with limited verbal communication. Participants shared that though some body language could be captured on video, there were still elements were missed, such as whether the patient was making eye contact, managing their hygiene, or if they were twitching or shaking. Nonverbal communication was also important for providers to assess patient comprehension. A physician shared:

*There is a lot to be said about actually being able to see the person and see how they’re reacting nonverbally to what you’re saying to them. And then it also gives you a better idea, especially with the [IDD] population, of how much they’re registering what you’re saying to them. Whereas on the phone…you can just have the caregiver taking over and you have no idea if the person is even still there*.

This was even more challenging if patients, or their caregivers, were unable to set up their camera correctly or had insufficient internet quality to support a clear image.

Participants suggested that due to these limitations virtual care may be appropriate for simple issues but not for more complex issues where more nuanced communication is needed. A primary care physician explained:

*So if it’s a minor thing like we’re doing a [medication] refill and the patient’s completely stable and behaviourally stable and the patient has no concerns and the team has no concerns, then I think phone is great because it’s just more convenient for them than having to arrange to get the person here and everything. If it’s not straightforward and the person is doing worse or something’s changing or their behaviour is changing and they’re having symptoms, then I’d say the phone is a poor substitute and we really do need to see them to get all of those subtle sort of clinical cues of their body language and behavior and how they react.*

Another limitation of telephone visits was that providers were unable to use supplemental communication strategies such as images, gestures and other visual aids. A 35 year old woman with an intellectual disability and a hearing impairment explained, “*Because of my IDD, they use a lot of visuals [in-person] but over the phone they can’t really do that*.” Participants noted that video platforms could potentially offer greater flexibility to use images or videos to support communication, though none had experienced this yet.

Finally, telephone visits were challenging for people with IDD who had more trouble processing, understanding and remembering auditory information without accompanying visual cues. A 43 year old autistic woman shared “*If I’m on the phone, I’m probably using my weakest sense, which is my hearing. I’ve got good hearing, that’s not the problem. It’s recalling anything I’ve heard. Auditory I have trouble with.*”

Some participants, however, discussed that the lack of visual connection did not affect their ability to communicate. For example, a 33 year old man with an intellectual disability shared that in his experience with telephone appointments, “*it was easy to understand the doctor*.” A 29 year old autistic woman explained that except for instances where a visual was needed as part of a clinical exam, she preferred telephone appointments: “*If there’s no particular reason to be doing video, I mean, sometimes they want to see things, but if there’s no need for there to be video, I prefer to just do phone.*”

#### Connection and trust

The final theme was related the affect of virtual care on the ability of the patient, caregiver and health care provider to connect and build the trust necessary for effective communication. Some patients and caregivers found it more challenging to feel comfortable and establish trusting relationships through virtual interactions, especially with new health care providers, and were therefore less likely to disclose sensitive information. Some participants found that it was easier to form connections over video than telephone. For example, a 30 year old autistic woman shared that she has trouble recognizing people without a visual:

*I would have preferred to see who I was talking to rather than just hearing a voice, because just hearing a voice, I had to guess. OK, they said, ‘hi, it’s Dr. so-and-so’, but I would always have to like program in my brain to be like, oh, OK, so Dr. so-and-so is going to be calling me. […] My brain has to really […] memorize the voice, […] I had to really think about who it was. So… and then plus with seeing no number or no name, it’s even harder.*”

For others, video still could not replicate in-person connections. The mother of a 28 year old man with Down syndrome explained that it is difficult for her son to really connect with people unless he can interact with them in-person: “*I think part of it is he doesn’t get the same vibes from a computer. […] It’s almost like you’re one dimensional if you’re on the computer. Whereas when you’re in person, whether you’re a doctor or a nurse or a technician, he forms a little bit of a different connection with you.*”

Establishing rapport virtually could also be challenging for the caregiver, impacting how they share information about themselves with providers. The mother of a 29 year old autistic man explained:

*There’s something about the…relational piece of virtual that doesn’t lend itself to me being maybe more vulnerable about how I’m doing. As his primary care provider, am I going to say in a virtual call, ‘Dr. [removed], by the way, I recently received my own diagnosis of such and such’? I’m probably not going to do that so he’s not going to be aware that I’m actually supporting [my son] and have a health concern myself*.”

Conversely, some participants felt more comfortable interacting with providers, even new providers, virtually. For example, a 43 year old autistic woman shared:

*I like people, don’t get me wrong, but sometimes I have issues interacting with, especially strangers, in that kind of environment. [Virtual care] would give that physical barrier of distance so I can gauge my own...I’ll be comfortable where I am and can be a little bit more relaxed. […] I know the doctor’s office is safe but try telling my inner self that some days. […] Meeting someone the first time, remote sounds… sounds appealing*.”

Like this autistic woman, some participants were more comfortable in their own space with some physical distance between themselves and the provider and therefore better able to build connection.

## Discussion

This study investigated how virtual delivery of primary care affects communication between patients with IDD and their primary care providers. We identified four elements of communication that were affected: patient *participation and engagement* in the virtual appointment; the ability to *hear and be heard*, including hearing other participants during the health care visit and having the time and space to be heard; the ability to *see and be seen*, including the use of body language, facial expressions and visual aids to support communication; and the ability to build *connection and trust* between providers, patients and their caregivers.

In many cases, the virtual platform hindered these elements of communication. In alignment with previous studies conducted with the general population [28, 30, 33, 52], the telephone was found to be the most limited modality to support high quality communication. Although telephone communication was adequate or even preferred in some cases, it lacked a visual component to support nonverbal communication, facilitate turn-taking in appointments with multiple participants, and build connection. Nonverbal communication has been identified as particularly important for people with IDD [18] and in some cases, this resulted in patients with IDD being excluded entirely from the health care visit. Video was often an improvement over telephone, though as has been found in previous studies [29], nonverbal communication was still sometimes limited by technical challenges. That said, for people who need to see full facial expressions to aid with comprehension or comfort, video may be an important option if masks are required for in-person visits.

Overall, this study found that communication quality in virtual care for people with IDD was variable and the appropriateness of virtual care was dependent on the needs, capacities and experience of the individuals involved and the nature of the specific health care appointment. Similar to prior research with the general population [34, 52], study findings suggest that virtual visits may be less appropriate when seeing new providers and for more complex or sensitive issues. They are also less appropriate for people with IDD who require in-person interactions to fully develop relationships and engage in the health care interaction. Virtual visits can be particularly important to support positive communication for adults with IDD who find attending health care visits to be overwhelming or distressing. An important next step will be to develop guidelines for clinicians on how to work with patients and caregivers to determine when virtual care is appropriate and create an individualized communication plan. As a starting point, we are in the process of adapting existing patient communication tools to allow patients to inform providers of their preferred modality for healthcare visits (available at https://ddprimarycare.surreyplace.ca/).

This study was conducted in a context in which providers, patients and caregivers may have had relatively little experience with virtual care. Prior studies with the general population have shown that with more experience and training on how to use virtual platforms, patients, caregivers and providers report improved communication and rapport [25, 28, 53, 54]. Additionally, some of the challenges identified in this study were related to poor infrastructure to support virtual care, including issues with internet quality leading to distorted or choppy video and inconsistent functionality of virtual platforms (e.g., availability of chat box feature). These limitations may help explain why the vast majority of virtual care in Ontario has been delivered by telephone [31], despite the many advantages of video identified in this study. It is possible that with improved infrastructure and greater experience using virtual care, communication quality may improve.

More research is needed on specific strategies or techniques that clinicians can use to improve quality of communication in virtual encounters with patients with IDD so they can be optimized for those patients who can benefit from them. Health care providers also require improved training in effective communication for patients with IDD regardless of modality used. This includes how to: engage in triadic communication including the patient and their caregiver, assess patient comprehension, navigate patient tendencies towards acquiescence, use appropriate language complexity, and use assistive communication devices when appropriate (e.g., visuals, communication mats, easy read materials) [18, 21, 55]. Without these communication skills, providers will not be able to support effective communication in-person or virtually.

### Strengths and limitations

This is one of the few studies on virtual health care communication for patients with IDD. A strength of the study is the inclusion of diverse perspectives from patients, family members, IDD support staff and physicians; however, limited demographic data was collected and we cannot speak to participant diversity in terms of race and other intersectional identities. This study reflects experiences in one Canadian province and it is possible that patients in other jurisdictions with different infrastructure or training may have difference experiences. Many of the primary care physicians who participated in this study had practices with a particular focus on patients with IDD. Physicians with less experience with this patient population may have identified different challenges.

Due to COVID-19 restrictions, the interviews in this study were all conducted by video or telephone. A benefit of this approach was that it facilitated participation from individuals across a large geographic region, however, though there were no requests for an in-person option, it is possible that this limited who was able or willing to participate. Prior studies have supported the validity of remote interviews in qualitative research [56, 57] and participants in this study generally provided positive feedback about the interview experience. However, some interviews were disrupted by technical challenges and it is possible that these or other issues we were not aware of (e.g., lack of rapport or participant discomfort) impacted the information shared in the interviews. The aim of this study was to elucidate the range of ways in which virtual delivery may impact communication for this heterogeneous patient population; the study design did not allow for conclusions to be drawn on the experiences of specific sub-populations. It will be important to explore these nuances in future research.

## Conclusion

Effective communication is a critical component of high quality primary care and is a long identified challenge for patients with IDD. This study found that there are some patients with IDD for whom virtual care can support higher quality communication by allowing them to participate from spaces where they feel more comfortable and can better engage in the health interaction. There were also many patients with IDD for whom virtual care made communication more challenging. Some of these challenges seem to be innately tied to the nature of virtual interactions and there is likely a segment of the IDD population who will always require in-person care to support high quality communication. However, other challenges were due to poor infrastructure to support virtual care and providers, patients and caregivers lacking the necessary skill and experience to optimize virtual care. It is possible that with improved infrastructure, experience and training, quality of communication could improve. This study suggests that a flexible patient-centred approach to care delivery is needed that includes in-person, telephone and video options based on patient need.

## Data Availability

The datasets generated and analysed during the current study are not publicly available due to confidentiality concerns, but the de-identified data are available from the corresponding author on reasonable request.

## List of abbreviations

IDD: Intellectual and developmental disabilities
